# An Improved Phenotyping Protocol for Panama Disease in Banana

**DOI:** 10.3389/fpls.2019.01006

**Published:** 2019-08-06

**Authors:** Fernando A. García-Bastidas, Alexander J. T. Van der Veen, Giuliana Nakasato-Tagami, Harold J. G. Meijer, Rafael E. Arango-Isaza, Gert H. J. Kema

**Affiliations:** ^1^Laboratory of Plant Breeding, Wageningen University and Research, Wageningen, Netherlands; ^2^Laboratory of Phytopathology, Wageningen University and Research, Wageningen, Netherlands; ^3^Escuela de Biociencias, Universidad Nacional de Colombia, Medellín, Colombia

**Keywords:** Panama disease, TR4, *Fusarium oxysporum* ff. spp. *cubense*, mung bean, microconidia, phenotyping, spore production

## Abstract

*Fusarium oxysporum* (*Fo*) belongs to a group of soil-borne hyphomycetes that are taxonomically collated in the *Fusarium oxysporum* Species Complex (FOSC). Hitherto, those infecting bananas were placed in the forma specialis *cubense* (*Foc*). Recently, however, these genetically different *Foc* lineages were recognized as new *Fusarium* spp. placed in the Fusarium of Banana Complex (FOBC). A member of this complex *F. odoratissimum* II-5 that uniquely comprises the so-called Tropical Race 4 (TR4), is a major problem sweeping through production zones of Cavendish banana in several regions of the world. Because of this, there is an urgent need for a phenotyping method that allows the screening for resistance to TR4 of large numbers of banana genotypes. Most Fusarium species produce three types of spores: macroconidia, microconidia and the persistent chlamydospores that can contaminate soils for many years. Inoculum production has been an important bottleneck for efficient phenotyping due to the low or variable number of conidia and the elaborate laboratory procedures requiring specific infrastructure. Here, we report a rapid, simple and high-yielding spore production method for nine *F. oxysporum* formae speciales as well as the biocontrol species *Fo*47 and *Fo*618-12. For *Fusarium* spp. causing Fusarium wilt or Panama disease of banana, we used the protocol for four species comprising the recognized physiological races, including Tropical Race 4 (TR4). We subsequently tested the produced inoculum in comparative inoculation trials on banana plants to evaluate their efficiency. All assays resulted in typical symptoms within 10 weeks; significant differences in final disease ratings were observed, depending on inoculum concentration. Pouring inoculum directly onto banana plants showed the most consistent and reproducible results, as expressed in external wilting, internal discoloration and determined by real-time PCR assays on entire rhizomes. Moreover, this method allows the inoculation of 250 plants per hour by one individual thereby facilitating the phenotyping of large mutant and breeding populations.

## Introduction

The genus *Fusarium* comprises many of the most important fungal plant pathogens. It is ranked fifth on a list of top fungal plant pathogens based on scientific and economic importance (Ploetz, 2005b; Dean et al., 2012). The *Fusarium oxysporum* species complex (FOSC) combines a morphologically diverse suite of species, including plant pathogens, saprophytes and even facultative human pathogens (Dean et al., 2012). Many of the pathogens cause wilting diseases, root rots and damping-off in hundreds of plant species (Domsch et al., 1980; Gerlach and Nirenberg, 1982; Nelson and Toussoun, 1983; Meldrum et al., 2012). Over 120 formae speciales (ff. spp.) have been described (Baayen et al., 2000; Hawksworth, 2001) each affecting one or a limited number of different host plant species. Differences in pathogenicity on specific host cultivars define physiological races among isolates, which has been studied intensively in some pathosystems (Kistler, 1997; Baayen et al., 2000; Takken and Rep, 2010; Meldrum et al., 2012).

*Fusarium oxysporum* f. sp. *cubense* (*Foc*) is the hitherto species names of strains infecting banana and causing Fusarium wilt, or Panama disease (*Musa* spp.). However, it has long been recognized that *Foc* has a polyphyletic origin (O’Donnell et al., 1998; Ploetz, 2005b; Lievens et al., 2009), hence comprises a suite of genetically distinct lineages (Ordonez et al., 2015b). Therefore, Maryani et al. (Maryani et al., 2019) have recently revised the taxonomy of *Foc* and designated different species names to strains affecting banana and merged them into the Fusarium of Banana Species Complex (FOBC). The disease cycle of these *Fusarium* spp. starts with infection of the root system and subsequent colonization of the vascular tissue, leading to water stress, severe chlorosis and wilting (Beckman, 1987; Ploetz, 2015). Infected plants frequently die before they produce bunches, hence Fusarium wilt significantly reduces yields in infested fields (Stover and Ploetz, 1990; Dita et al., 2010). Race 1 strains caused one of the worst botanical epidemics in history and decimated the commercial Gros Michel banana based industry in Central America in the 1950s (Ploetz, 2005a). As a result, Gros Michel was replaced with resistant and now globally cultivated Cavendish clones. Albeit that these quenched the race 1 driven epidemic, many regionally important banana varieties are still susceptible to these strains and succumb to the disease (Ploetz, 2006). Meanwhile, another species, *F. odoratissimum* II-5, that uniquely comprises the so-called Tropical Race 4 (TR4), is sweeping through major production zones of Cavendish banana (Butler, 2013; García-Bastidas et al., 2014; Ordonez et al., 2015a; Zheng et al., 2018), thereby significantly affecting susceptible local and regionally important banana varieties destined for domestic markets (Ploetz, 2015).

For most crops, host resistance is a cornerstone for sustainable disease management, usually achieved by intensive breeding programs. However, breeding for resistance in banana is limited and has not resulted in diversification. Cavendish is the cornerstone for the international trade and hence, TR4 threatens the entire global production. Improved banana varieties are mostly mutants of Cavendish clones that were selected in extensive field trials. These are time consuming, expensive and sometimes unreliable due to variable environmental conditions and unknown inoculum diversity and distribution (Mert and Karakaya, 2003; Subramaniam et al., 2006; Sutanto et al., 2013). In contrast, greenhouse phenotyping facilitates higher throughput under controlled conditions with specific fungal genotypes, leading to more reproducible results, which accelerate breeding programs (Smith et al., 2008). With the progress in high-throughput genotyping methods, phenotyping has become a major bottleneck for plant improvement, particularly for perennial crops such as banana.

Therefore, efficient inoculum production is the first critical factor in optimizing phenotyping protocols. For *Fusarium* spp. several protocols were developed (Sun and Su, 1984; Adesemoye and Adedire, 2005; Amorim et al., 2009; Dita et al., 2011; García-Bastidas et al., 2014; Li et al., 2014; Ordonez et al., 2015a), of which many are based on the use of commercial growth media but also natural sources such as beans (*Vigna radiata* L.) (Bai and Shaner, 1996; Li et al., 2001; Yuan and Zhou, 2005; Mudge et al., 2006; Amorim et al., 2009; Dita et al., 2010; Dita et al., 2011; García-Bastidas et al., 2014; Li et al., 2014; Ordonez et al., 2015a). However, these methods cannot be up scaled to the large volumes of inoculum required for extensive phenotyping experiments (Burgess et al., 1991; Leslie et al., 2006), due to either large quantities of expensive culture medium or costly infrastructure. Moreover, the procedures are very labor intensive. Hence, these practical constraints have contributed to manifold inoculation assays (Sun and Su, 1984; Smith et al., 1999; Mohamed et al., 2001; Subramaniam et al., 2006; Smith et al., 2008; Wu et al., 2010; Dita et al., 2011), lacking uniformity, which complicates data comparison and interpretation. Thus, there is a need for a standardized and widely accepted phenotyping protocol to evaluate Panama disease resistance in banana.

Hitherto protocols facilitated the mere screening of approximately 15 plants hour^–1^ person^–1^ (Dita et al., 2010; Dita et al., 2011; Ordonez et al., 2015a). Clearly, this hampers throughput and potential automation during phenotyping mutant panels or segregating populations that usually comprise hundreds or even thousands of plants. Here, we report the development of an optimized mung bean-based *Fusarium* spp. inoculum production protocol that matches all the aforementioned constraints and suffices for screening up to 250 banana plants per hour per person. Moreover, its wide applicability was shown for other *Fusarium* spp. affecting different crops.

## Materials and Methods

### *Fusarium* spp. and Growth Conditions

In total, eleven *Fusarium* spp. genotypes were tested, including the known races: race 1 (R1), Race 2 (R2), Subtropical Race 4 (ST4) and TR4, as well as two well-known *Fo* biocontrol agents (Table 1). All strains are maintained in the Wageningen University and Research (WUR) collection and mostly originate from infected plant tissues. Strains were grown at 27–28°C on potato dextrose agar (PDA; Sigma Chemical Co., St. Louis, MO, United States) for 5 days in the dark and plugs were then taken from the edge of the colony to inoculate liquid media.

**TABLE 1:** Origins and characteristics of the *Fusarium* spp. strains used in this study.

***Fusarium* spp.^1^**	**Code**	**Host**	**VCG^2^**	**Origin**	**Provider**
FOSC clade 4	*Foc* R1	Banana	Unknown	Cruz das Almas, Bahia (Brazil)	M. Dita, Netherlands
*F. tardichlamydosporum*	*Foc* R2	Banana	0124	United States	K. O’Donnell, United States
*F. odoratissimum* II-5	TR4	Banana	01213	Indonesia	R. Ploetz, United States
*F. phialophorum*	*Foc* ST4	Banana	0120	Canary Islands (Spain)	J. Hernandez, Spain
*F. oxysporum* f. sp. *melonginae*	*Fom*	Eggplant	n.a.	Israel	Unpublished
*F. oxysporum* f. sp. *lycopersici*	*Fol*	Tomato	n.a.	Netherlands	Unpublished
*F. oxysporum* f. sp. *cepae*	*Foce*	Onion	n.a.	Australia	Unpublished
*F. oxysporum* f. sp. *gladiola*	*Fog*	Gladiola	n.a.	–	Unpublished
*F. oxysporum* f. sp. *albedinis*	*Foca*	Date palm	n.a.	Canary Islands (Spain)	Unpublished
FOSC Clade 3	*Fo*47	Biocontrol	n.a.	France	Lemanceau et al. (1991)
–	*Fo*618-12	Biocontrol	n.a.	Netherlands	Postma and Luttikholt (1996)

*^1^Names adapted based on latest classification on Maryani et al. (2019). ^2^VCG, vegetative compatibility group; *Fo, Fusarium oxysporum.**

### Plant Material

*In vitro* plants were obtained from various sources (Supplementary Table 1), transferred to individual 1L pots containing a standard soil from the WUR Radix, Unifarm greenhouse facility (Swedish sphagnum peat 5%, grinding clay granules 41%, garden peat 5%, beam structure 4%, steamed compost 33%, PG-Mix-15-10-20-12%) and then placed in an environmentally controlled greenhouse compartment (28 ± 2°C, 16 h light, and ∼85% relativity humidity). Plants were acclimatized under plastic for 2 weeks to maintain high humidity conditions and thereafter grown for ∼2.5 months prior to inoculation. We used Cavendish “Grand Naine” for all comparative analyses but added additional banana accessions with various levels of resistance to TR4 for validation.

### Spore Production

To determine the optimal conditions for conidia production we used the reference *F. odoratissimum* II-5 TR4 isolate II-5 (Dita et al., 2010) as it is currently the most important threat to global banana production. Six sporulation media (SM) were prepared by autoclaving 500 ml water in 1 L Erlenmeyer flasks supplemented with 20 gr pre-boiled Mung beans, as in the original protocol (SMB20; Bai and Shaner, 1996), or 20 gr (SMF20), 5 gr (SMF5), 2 gr (SMF2), 1 gr (SMF1), or 0.5 gr (SMF05) of fresh Mung beans, respectively. The Erlenmeyer’s were closed with cotton plugs and sterilized at 120°C for 20 min. Five mycelium plugs were taken from a freshly grown PDA plate and were transferred to the Mung bean media. The inoculated Erlenmeyer flasks were incubated on a rotary shaker at 130 rpm at 25 ± 2°C for a maximum of 8 days. A 1 ml aliquot was taken under sterile conditions and passed through two layers of sterile cheesecloth to remove hyphal fragments at 1, 3, 6, and 8 days after inoculation (dai) for viability testing and spore quantification. A 10 μl sample of the remaining suspension was plated on a PDA plate and then incubated at 25°C in the dark to germinate the spores after which the growing area was measured. The number of conidia was counted using Glastic Slides (Hycor Biomedical., CA, United States), photographs of each grid were taken with a light microscope (Zeiss Axiocam ICc3) and spores were counted manually, using a hemocytometer, with five repetitions per sample. All experiments were repeated twice. After evaluations of the different media, SMF2 was used for spore production of all other *Fusarium* spp. infecting banana (Table 1) with quantification of spore concentration at 6 dai.

Chlamydospores of TR4 were produced following the protocol of Amorim et al. (2009) with minimal modifications. Briefly, plugs with mycelial growth of *Foc* were mixed with twice autoclaved substrate composed by sandy soil, corn powder and distilled water in 500 ml Erlenmeyer flasks. Then flasks were incubated at 25°C for around 12 days. New autoclaved sandy soil was infected with the infected substrate in relation 1:12, then flasks with the mixture were incubated for an additional 6 weeks. Infected maize kernels were produced by inoculating 100 g of sterilized grains in Erlenmeyer flasks with five TR4 plugs derived from a fresh growing colony on PDA plate. Flasks were incubated at 25°C in the dark for 10 days.

### Inoculation and Plant Maintenance

Five phenotyping methods were compared: (i) dipping banana plants with trimmed roots in spore suspension and transplanting them into either non-sterilized soil (*dipping soil*) or (ii) sterilized sand (*dipping sand*), (iii) uprooting banana plants and transplanting them into soil infested with chlamydospores (*chlamydospore method*), (iv) adding TR4 colonized maize kernels to soil of potted banana plants (*kernel method*), and (v) pouring inoculum directly on the soil of potted banana plants (*pouring method*). For the dipping methods with transplanting either to soil or sand, banana plants were uprooted and the root systems were rinsed with water and then trimmed to a third of the original mass (removing ∼10 cm from the tip of the root) and left in water to avoid plant stress until inoculation before immersing them for 30 min in inoculum of various concentrations (10^3^, 10^4^, 10^5^, 10^6^ spore ml^–1^). For the chlamydospore method, banana plants were transplanted in fresh soil that was mixed with approximately 10^4^ chlamydospores.gram of soil^–1^ at various ratios (2,5; 5; 10, and 20 g L^–1^). For the maize kernel method, 3, 5, 10, or 20 colonized grains were placed in two equidistant holes from the base of the banana plant. Finally, for the pouring method, 200 ml of inoculum with various concentrations (10^3^, 10^4^, 10^5^, 10^6^ spore ml^–1^) were directly added onto the pots. For all experiments, we selected five plants with 6/7 leaves and 30/50 cm height which were maintained in a closed pot system to prevent inoculum leakage and cross contamination (Mohamed et al., 2001) and for each treatment, non-inoculated Cavendish “Grand Naine” banana plants were used as mock. The resistant accessions “Pahang” and cv. Rose were included as negative controls.

### Disease Assessment

Upon infection, the plants were monitored and scored for disease symptoms and progress at weekly intervals. The latency period was set as the time elapsed between inoculation and the appearance of the first symptoms in three out of five plants. Plants were externally and internally inspected when totally decayed or when 75% of the leaves turned yellow or ultimately at 10 weeks after inoculation (wai). External symptoms – the percentage of yellowing/wilting leaves – were scored following a 1–4 class scale in which 1 = 0 > *x* ≤ 25%, 2 = 25 < *x* ≤ 50%, 3 = 50 < *x* ≤ 75%, and 4 = 75 < *x* ≤ 100%. In addition, morphological changes of leaves and pseudostem splitting were recorded. For internal evaluation, plants were uprooted, cleaned and cut longitudinally at the rhizome (corm) of each plant. Disease severity was visually assessed following a 1–6 scale where 1 = No discoloration in the corm, 2 = isolated points, *x* < 5%, 3 = 5 < *x* ≤ 30%, 4 = 30 < *x* ≤ 50%, 5 = 50 < *x* ≤ 90%, and 6 = plant totally decayed *x* < 90%. To guarantee an accurate quantification of the affected and discolored tissues we conducted image analyses (ImageJ 1.49)^1^ and disease indexes were calculated following Mckinney (1923).

Finally, fungal biomass per individual corm was determined by qPCR. Corms were sliced into small pieces (3–5 cm) and collected in 50 ml tubes and freeze-dried for 48 h (Epsilon 1-4 LSC, Christ GmbH, Germany). The remaining mass was weighed after which three chrome-steel beads (6.35 mm, Biospec) were added and the samples were ground using a conventional vortex (IKA Labortechnik, Staufen, Germany) until the material was homogenized (∼1,5 min). Hundred mg tissue was processed for DNA extraction using a Kingfisher robot (Thermo Labsystems, Finland) and the AGOWA Sbeadex^®^ Maxi plant DNA isolation kit (LGC Genomics, Germany). Samples were mixed with 600 μl lysis buffer, bead beaded (Bertin technologies, Ampere montigny-le-Bretonneux, France) for 40 s and then incubated at 65°C for 15 min, followed by centrifugation for 20 min at maximum speed in an Eppendorf centrifuge (Eppendorf 5415D, Germany) after which 200 μl of supernatant per sample was collected and transferred to a deep 96-well plate containing 520 μl binding buffer following the manufacturer’s instructions.

The total amount of genomic DNA was quantified by PicoGreen using 5 μl of the total DNA that was placed in a deep 96-well plate with 100 μl of 1× PicoGreen and 99 μl TE after which the samples were measured with Tecan Infinite M200Pro using Icontrol 107 software (Morrisville, NC, United States). Verifications TR4 infections were performed by multiplex PCR using the *EF* and *BanActin2* genes as internal controls (Dita et al., 2010) and quantitative PCR (qPCR) by SYBR^®^ Green technology using the commercial ClearDetections^®^ TR4 molecular diagnostic kit (Clear^®^ Detections, Wageningen, Netherlands) in a real time PCR machine (ABI 7500, Thermo Fisher Scientific, United States). The amount of biomass was calculated as the amount of DNA.mg-1 dry weight based on the resulting Ct values using a 10-fold dilution series of TR4 genomic DNA as standard (1 ng–1 fg).

### Statistical Analysis

The comparative inoculation assay experiment was set up in a factorial design with two factors: method and level of inoculations. Means of the percentages of chlorotic foliage, corm discoloration, disease index, corm dry weight and Ct values obtained by real time PCR were analyzed by ANOVA and differences of the means of each variable were compared by using Fisher’s test (LSD, *p* = 0.005).

## Results

### *Fusarium* spp. Inoculum Production Depends on Mung Bean Medium Composition

**FIGURE 1:**
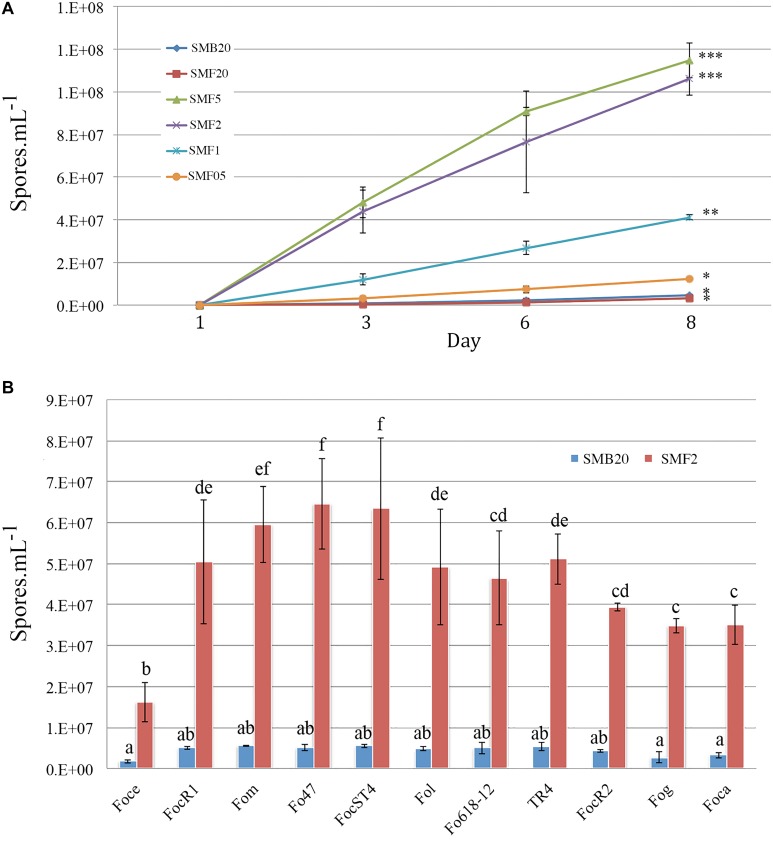
Conidia production of *Fusarium* spp. in sporulation media (SM). **(A)** Spore production over time for Tropical Race 4 (*F. odoratissimum* II-5) in different media. **(B)** Spore production in SMB20 and SMF2 for 11 *Fusarium oxysporum* ff. spp. Spores were quantified after 6 days (*n* = 3) and the experiment was repeated at least twice (*P* > 0.05, treatments with the same letter/symbol are not significantly different).

Different amounts of Mung beans in gr per 500 ml water were tested in order to standardize the most suitable sporulation media (SM). Conidia production of *F. odoratissimum* II-5 (TR4) commenced within 24 h after inoculation on the media. All SM, except SMF20 (20 g), showed a significant increase of conidia compared to the control SMB20 (original protocol) (Figure 1A). After 24 h, a steep increase in conidia production was observed in SMF5 (5gr), SMF2 (2gr), and SMF1 (1gr), while a moderate increase was observed in SMF05 (0,5 gr). In contrast, no conidia were observed for SMB20 and SMF20 at that time. During final quantification at 8 dai, the SMB20 culture contained 4.5 × 10^6^ conidia ml^–1^ while the highest concentrations were obtained in SMF5 and SMF2, with over 1.1 × 10^8^ conidia ml^–1^ (Figure 1A). Albeit that SMF5 and SMF2 excelled in spore production at 8 dai, the former was eventually chosen as the final filtering and dilution of conidia was much easier due to the higher content of mycelia in SMF5.

### The New Mung Bean Protocol Is Applicable for Other *Fusarium* spp.

**FIGURE 2:**
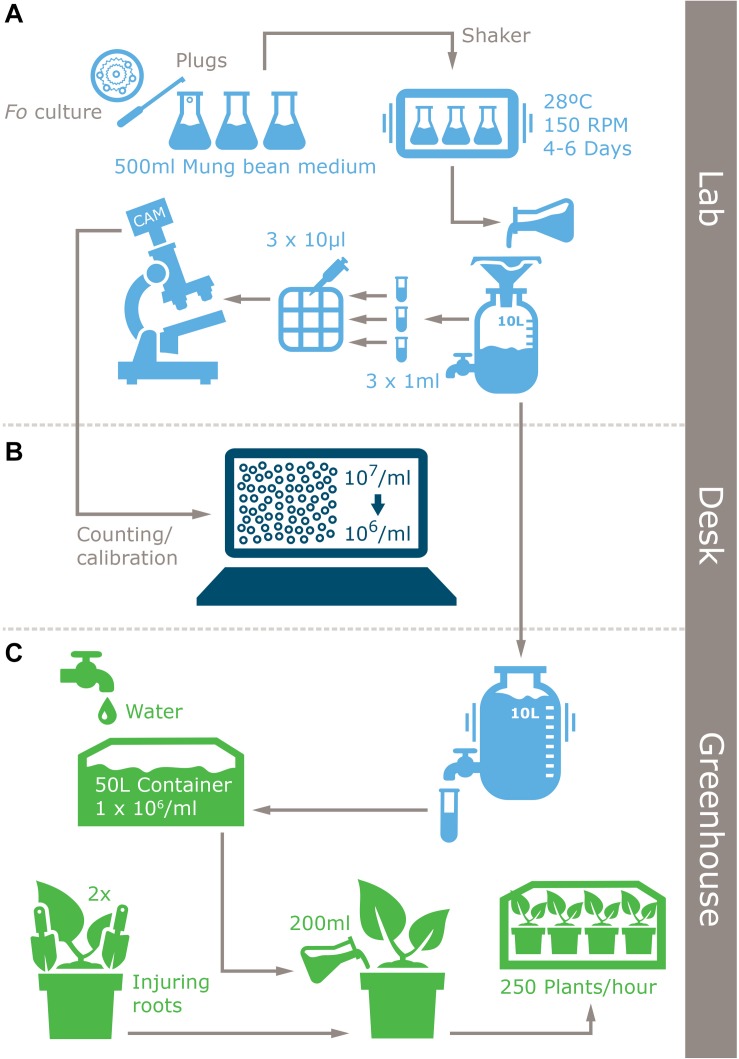
Infographic of the improved inoculum production protocol for *Fusarium* spp. and plant inoculation method. **(A)** Laboratory inoculum production, **(B)** inoculum quantification, and **(C)** inoculation.

To test whether the SMF2 protocol is applicable for other *Fusarium* spp. and two *Fo* biocontrol strains (Table 1) we compared spore production with SMB20 (Figure 1B). All strains produced significantly more conidia in SMF2 than in SMB20 media. Despite the fact that *Foce* was the least productive (1.6 × 10^7^ spores ml^–1^), SMF2 produced 10-fold the quantity over SMB20. The highest concentration of conidia was obtained for *Fom, Fo*47 and ST4 reaching > 6.0 × 10^7^ spores ml^–1^ (Figure 1B), but TR4 produced more than 5.0 × 10^7^ spores ml^–1^ (Figure 1B), which was sufficient to inoculate 250 plants per hour by one person (see infographic Figure 2).

### Comparison of Inoculation Assays and Inoculum Concentrations

**FIGURE 3:**
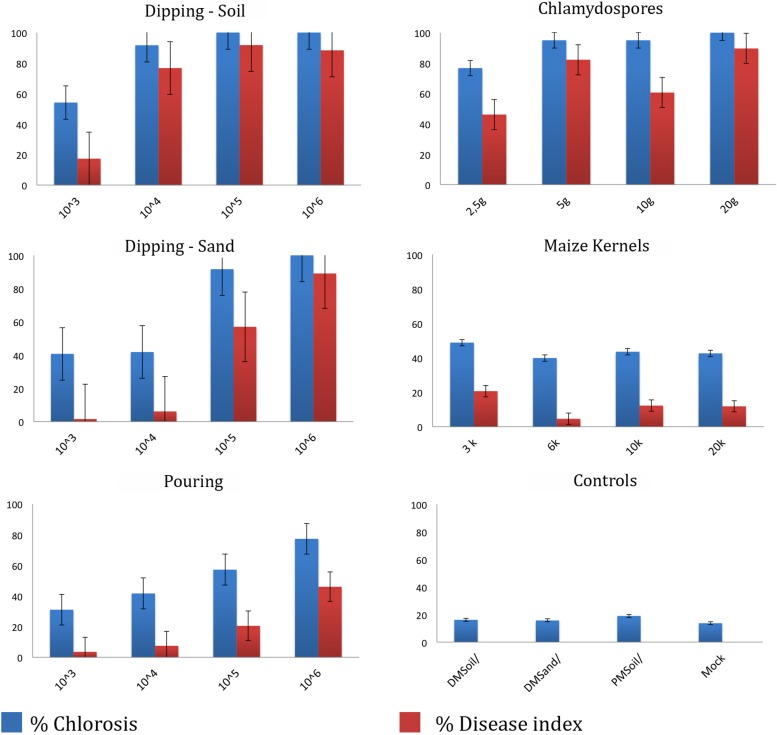
Dose-response disease indexes of “Grand Naine” plants at final score (5–10 weeks after inoculation) with *Fusarium odoratissimum* II-5 (Tropical Race 4) using five inoculation methods. For each treatment the average result for four inoculum doses are expressed as percentage of foliar chlorosis and as the disease index at the time of scoring.

To determine the most optimal inoculation method we compared five commonly used protocols (Figure 3). Initial chlorosis/wilting was observed for the dipping and chlamydospores inoculation methods, as well as their un-inoculated controls but these recovered after 2 weeks suggesting that these effects resulted from root trimming. Depending on the method used, the latency period was between 2 and 4 weeks after inoculation (wai), but was shortest in plants inoculated with the highest inoculum concentrations and using inoculation methods that involved root trimming (e.g., dipping and chlamydospore methods). The latency period for the pouring method was approximately 3 wai at all inoculum levels, whereas the application of colonized kernels resulted in a significantly delayed latency period (Supplementary Table 2). Plants challenged with high inoculum concentrations decayed 5–7 wai, except for the maize kernel treatment, which showed inconsistent values between low and high inoculum concentrations. All controls showed natural chlorosis and senescence and hence had score 1. Plants scored 2 once low inoculum concentrations were used for the dipping (sand) and pouring methods, as well as after using the maize kernels assay. The dipping (sand) and pouring methods with 1 × 10^4^ spores ml^–1^ consistently scored 3, whereas all other treatments resulted in score 4 for foliage discoloration.

**FIGURE 4:**
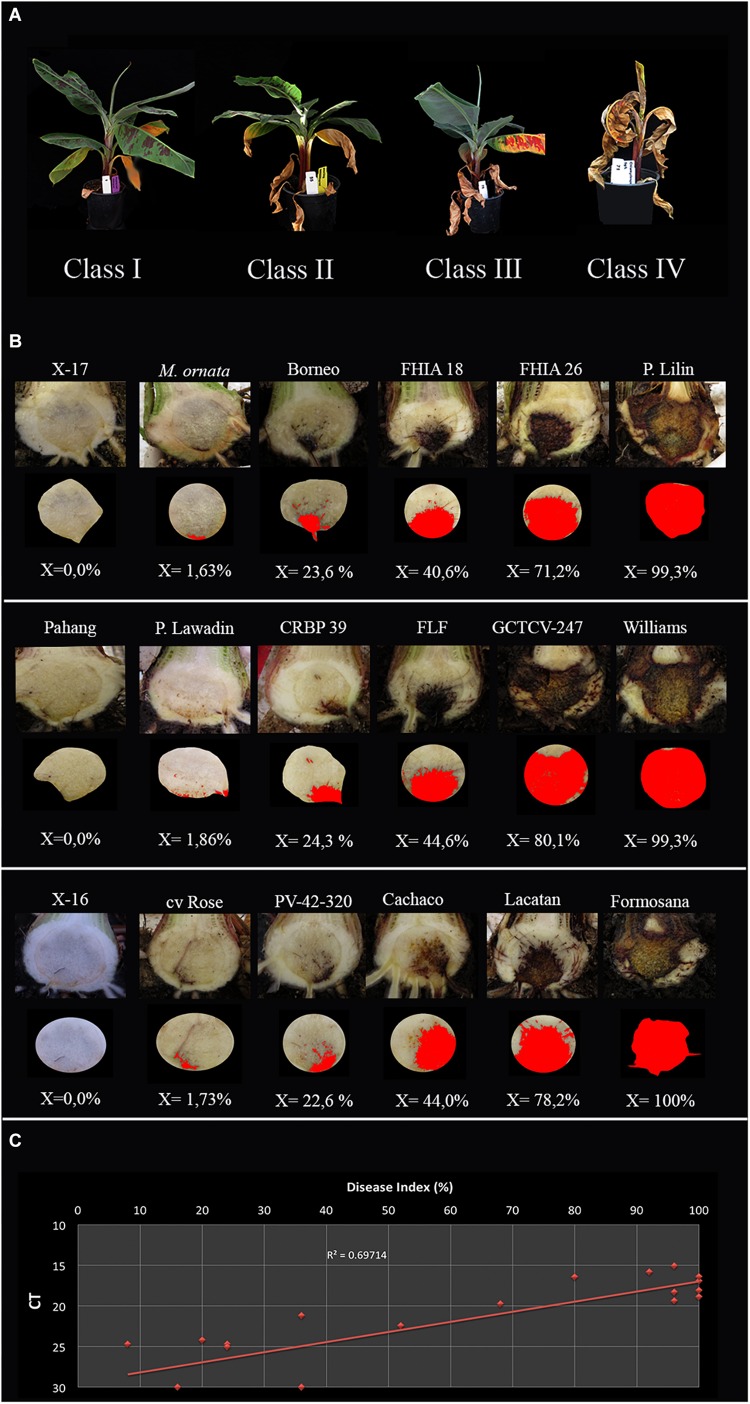
Panama disease progress incited by Tropical Race 4 (*Fusarium odoratissimum* II-5). **(A)** Four class rating scale of leaf chlorosis: I = (0 > *x* ≤ 25%), II = (25 < *x* ≤ 50%), III = (50 < *x* ≤ 75%), and IV (75 < *x* ≤ 100%); **(B)** Internal severity levels of 18 banana accessions (Supplementary Table 2) and the accompanying percentages of affected tissue as calculated by ImageJ; **(C)** The correlation between qPCR quantification and disease index per corm with trendline and *R* value.

**FIGURE 5:**
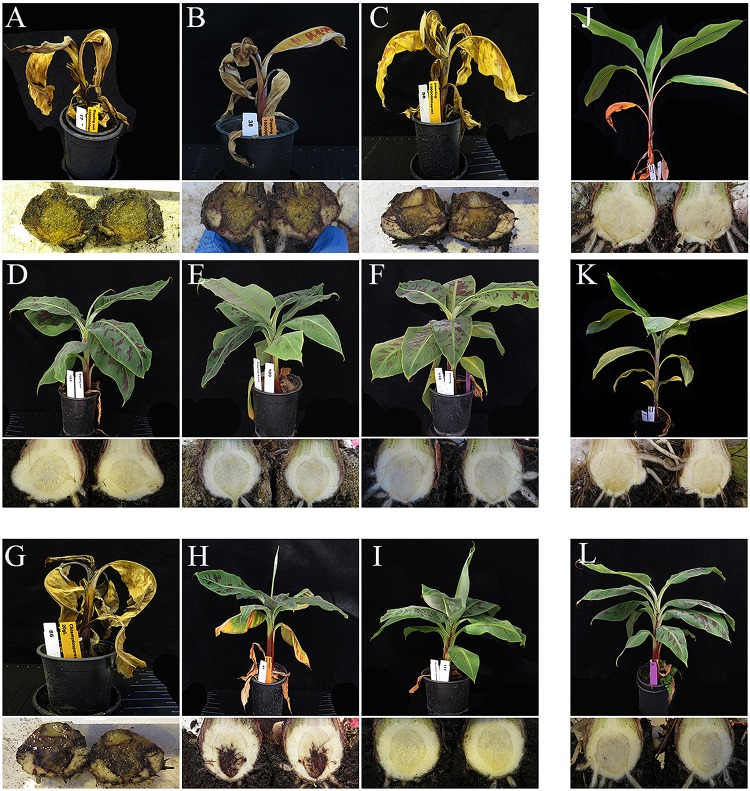
External and internal symptoms of ‘Grand Naine’ at six weeks after inoculation with *Fusarium odoratissimum* II-5 (Tropical Race 4). The panels **(A–F)** show the results of different inoculation methods (top panels); **(A)** DM soil, **(B)** DM Sand, **(C)** pouring method and their respective controls in panels **(D–F)**. Panels **J,K** are the associated negative controls using resistant accessions cv. Rose **(J)** and ‘Pahang’ **(K)**. Plants inoculated with chlamydospores **(G)** or Maize kernels **(H)**, also developed similar symptoms but the latency period differs from that of conidial inoculations. Plants shown were challenged with the highest inoculum doses described for each method. The negative controls were ‘Grand Naine’ inoculated with race 1 using the dipping method **(I)** and the mock **(L)**.

All inoculation assays resulted in internal corm discoloration, but affected areas differed significantly between the applied methods and inoculum concentrations (Figures 4, 5 and Supplementary Table 2). Generally, higher inoculum concentrations resulted in higher disease severities, except for the maize kernel treatment, where inoculum dosage and symptom development were not significantly correlated. For the other assays, all inoculum concentrations resulted in > 75% corm discoloration. The determined disease indices (DI) showed a range of values that divide the inoculation assays into three groups (I-III) based on their severity (Figure 4 and Supplementary Table 2): maize kernel and dipping methods (10^3^ spores ml^–1^) and dipping (sand) (10^3^ and 10^4^ spores ml^–1^) resulted in low severities with DI values between 0 and 50; pouring (10^5^ spores ml^–1^) and chlamydospore (2,5g) treatments exhibited moderate severities with DIs between 50 and 80; all remaining treatments developed high severities with DIs between 80 and 100. Since the newly developed pouring assay displayed the widest variation in DI, we chose to validate this assay on 12 additional banana accessions with various levels of resistance to TR4 (Figure 4C). This enabled the ranking of these accessions by their DIs from immune to highly susceptible (Figure 4). Across all experiments, “Grand Naine” plants inoculated with race 1 (Figure 5F) as well as “Pahang” and “cv Rose” inoculated with TR4 did not develop any external and internal symptoms, independent of the used inoculum concentrations (Figures 5K,L). All water controls remained healthy (Figures 5G–J).

### Additional Corm Analyses

Corm dry-weights correlated with DIs, except for the colonized maize kernels assay (Supplementary Table 1). Lyophilizing complete corms was performed to avoid statistical errors in sampling and enabled the detection and quantification of TR4 biomass. Nearly all corms derived from infected plants tested positive for TR4 with conventional PCR (Dita et al., 2010), except some dipping (sand) and pouring treatments (both 10^3^ spores ml^–1^) and some of the pouring method (10^4^ spores ml^–1^) replicates. However, qPCR analysis confirmed the presence of TR4 DNA in all samples with average Ct values between 15.06 and 24.73, whereas all controls plants were negative (Supplementary Table 2), but the correlation with DI was rather low (*R*^2^ = 0.697, Figure 4D).

## Discussion

The global dissemination of plant pathogens and pests is a serious concern for future food and feed production (Bebber et al., 2014). Cereal plagues draw massive attention (Sun, 1978; Bhattacharya, 2017), but diseases in orphan crops usually pass unnoticed. Banana is no exception, as the occurrence of TR4 threatening Cavendish bananas in Taiwan was already noticed in the 1960s and first published in 1978 (Sun, 1978), the occurrence in Jordan and other countries outside South East Asia drew eventually global attention (Butler, 2013; García-Bastidas et al., 2014; Ordonez et al., 2015a). Since then, Panama disease is again considered a serious threat for global banana production, which results in an increased level of fundamental and applied research. Hence, there is an urgent need for reliable and standardized phenotyping protocols to seek banana accessions with adequate resistance and to verify the efficacy of control methods. Such assays should enable high throughput data gathering, ideally of parallel screens using multiple *Fusarium* strains, thereby facilitating comparisons of data collected in different laboratories.

Here, we describe an inoculum production protocol that entails efficiency, by eliminating pre-boiling and pre-filtering steps prior to autoclaving, and by using just 2 grams of Mung bean seeds to produce between 1-7.5 × 10^7^ spores ml^–1^ in 6 days, irrespective of the *Fusarium* species and more than 1 × 10^8^ at day 8 with TR4. Comparable results were also obtained for other *Fo* species, including the biocontrol strains *Fo*47 and *Fo*618-12. The production of 5 × 10^4^ to 3 × 10^6^ spores ml^–1^ was reported for *Rhyzopus oligosporus* using 100 gr L^–1^ of Mung bean sprouts (Nout et al., 1987) and Bai and Shaner (1996) found *F. graminearum* condia concentrations to oscillate between 4.6 to 5.5 × 10^5^ spores ml^–1^. Jo et al. (2015) compared seven sporulation media for *Fo* f. sp. *niveum* for watermelon bioassays and reported that in Czapek-dox broth 4.0 × 10^4^ spores ml^–1^ were obtained, whereas a maximum production of 2.6 × 10^7^ spores ml^–1^ was found in V8-juice broth. For race 1 (VCG01217), Subramaniam et al. (2006) reported a production of 1.4 × 10^5^ at 7 dai, 4.6 × 10^5^ at 21 dai and only 6.2 × 10^5^ spores ml^–1^ at 4 wai. The latter was confirmed as the production of SMB20 resulted in 8.3 × 10^5^ spores ml^–1^. Thus, our improved protocol allowed us to produce on average 100 times more spores than previously published methods, which will be beneficial for studying various *Fusarium* pathosystems and also boosts the production of biocontrol strains like *Fo*47 (Postma and Luttikholt, 1996). The production of spores in all media was reproducible, although some variation was observed in the final number of spores, potentially due to variations in the prepared media or the origin of isolates (Oswald, 1949; Nelson et al., 1994). The produced conidia were infective across the tested bioassays. We observed that optimal conidia production was accomplished at 6 dai since mycelial formation at later stages complicated inoculum filtering and eventually resulted in lower recovered spore concentrations, thereby reducing the efficiency of the protocol.

After establishing the optimal inoculum production protocol, we evaluated different Panama disease phenotyping assays, including a new method in which a conidial TR4 suspension is directly poured onto the soil. This new method resulted in typical disease symptoms comprising wilting, chlorosis, malformation of the emerging leaf, pseudostem splitting as well as discoloration of the rhizome and in severe cases plant death, similarly to the effects of the other treatments and corresponding to results shown in other studies (Smith et al., 2008; Amorim et al., 2009; Dita et al., 2010; Dita et al., 2011; García-Bastidas et al., 2014; Ordonez et al., 2015a). However, external symptom development, such as chlorosis of the foliage, is an unreliable parameter for disease evaluation, despite that it is a direct indication for the pathogen’s progress (Smith et al., 2008; Wu et al., 2010; Dita et al., 2011; García-Bastidas et al., 2014; Li et al., 2014). Clearly, latency period depended largely on inoculum concentrations with the shortest period for high spore concentrations using the dipping method in soil, sand and in the chlamydospore method (∼10–15 dai), as reported before (Mohamed et al., 2001; Smith et al., 2008). However, apart from the required large inoculum volumes, the time-consuming root trimming provokes stress, which results in morphological changes, including atypical chlorosis that is easily confused with initial Panama disease symptoms. The observed chlorosis of older leaves during the first week was therefore attributed to plant stress. This was confirmed by the appearance of comparable symptoms for incompatible interactions such as race 1 – “Grand Naine” and TR4 – “Pahang” and cv. Rose.

Different propagules and infectious structures revealed significant variation in DIs. Thus, infectivity depends on the composition of the inoculum, i.e., micro and macroconidia, chlamydospores or mycelium (Smith et al., 2008; Amorim et al., 2009), which was also observed in the tomato – *Fo* f. sp. *lycopersici* pathosystem (Cal et al., 1997). The dipping and chlamydospore methods invariably resulted in high disease severities at high inoculum concentrations, but for the maize kernel assay results were too variable across the used inoculum concentrations, probably due to the limited distribution of kernels/inoculum propagules in the pots and/or their position to nearby roots.

Inoculum concentrations affect the latency period, as observed by external discoloration of the foliage, as well as internal symptom severity. The most intense corm discoloration values and subsequent highest DIs were observed for the invasive methods (DI 80–100). Jo et al. (2015) found a DI ∼90 in the susceptible watermelon cv. Sugar baby by using the dipping method in a concentration of inoculum of 1 × 10^6^ spores ml^–1^. However, no significant differences were observed when inoculum concentrations were modulated between 1- 9 × 10^6^ spores ml^–1^. In our trials, dipping methods produced very low corm severities (1.5 and 6.1%) and low DIs (8 and 24) at low inoculum concentrations (10^3^ and 10^4^ spores ml^–1^) and transplanting in sterilized sand, likely due to escapes, suggesting a minimal required inoculum concentration. However, transplanting in non-sterilized soil resulted in enhanced disease development (17.3 and 76% for 10^3^ and 10^4^ spores ml^–1^, respectively). Whether this is due to microorganisms present in untreated soil is unknown, which underscores the need for studies focusing on the role of the microbiome in *Fusarium* spp. – banana interactions (Köberl et al., 2017).

Phenotyping protocols require efficiency, reliability and discriminative power. The invasive methods proved to be effective and reproducible, but require extensive plant pre-treatment, including root cleaning and trimming which may take up to 10 min per plant. Subsequently, plants must be immersed 30 min in large volumes of inoculum and then be transplanted to new pots. Albeit that chlamydospores are important under field conditions, their production takes up to 3 months and accurate quantification is challenging. Moreover, extra quarantine and safety steps are needed, as chlamydospores are extremely aggressive, even at low doses as observed in our trials. Hence, it is practically impossible to produce batches for individual *Fusarium* genotypes or species. Thus, these invasive methods hamper throughput required for large experiments, such as replicated interaction trials, segregating populations in genetic studies or the evaluation of mutant panels. We found that the pouring method enables the inoculation of 250 plants per hour, by a single individual, which is a huge improvement of throughput compared to any other method. Moreover, it produces dose dependent DIs, thereby providing an adequate discriminating protocol for disease ranking. An inoculum concentration of 1 × 10^6^ spores ml^–1^ is the most suitable and resulted in consistent data, which is a great advantage over erratic field trials. These may take over 9 months before disease expression, due to the mostly unknown distribution of inoculum in the soil (Sun and Su, 1984). Current phenotyping assays often rely on visual (external) scoring, which is straightforward but has severe limitations. Here, we complement such visual scoring with image analyses, which resulted in reproducible and objective data enabling standardization. The validation by real-time PCR for fungal biomass quantification in the corm showed a low correlation between the amount of fungal DNA and DI, which is most likely highly influenced by DNA degradation in the corm at late stages of infection. Therefore, we conclude that the DI, based on the quantification of corm discoloration is the most reliable method to assess disease severities in banana – *Fusarium* interactions.

Taken together we described an improved method for spore production for *Fusarium* spp. and compared five banana inoculation methods concluding that – contrary to all other methods – the pouring method enables the inoculation of a large number of plants, can be done by one person and yields a final disease index that is proportionate with the applied inoculum concentration.

## Data Availability

All datasets generated for this study are included in the manuscript and/or the Supplementary Files.

## Author Contributions

FG-B and GK contributed to the conception and design of the study. FG-B performed the experiments, and organized and analyzed the database. AV contributed with the evaluation of inoculation methods, and organized and analyzed the data. GN-T contributed with the conidia production experiments, and organized and analyzed the data. FG-B, GK, RA-I, and HM wrote the paper. All authors contributed to the manuscript revision, and read and approved the submitted version.

## Conflict of Interest Statement

The authors declare that the research was conducted in the absence of any commercial or financial relationships that could be construed as a potential conflict of interest.

## Footnotes

1 http://rsb.info.nih.gov/ij/
