# Supplementary material for: An Improved Phenotyping Protocol for Panama Disease in Banana
Source: Front Plant Sci. 2019 Aug 6;10:1006. doi: 10.3389/fpls.2019.01006 (PMC6691145; doi:10.3389/fpls.2019.01006)
Supplement: Supplementary file 1 [file Table_1.docx]

**Supplemental Table 1**. Banana accessions that were evaluated in the comparative inoculation trials

| **Genotype** | **Genome composition** | **Source** | **Habitus** |
| --- | --- | --- | --- |
| A-11 | AA | CIRAD | Hybrid |
| Borneo | AA | Bioversity | Wild |
| IND 110 cv Rose | AA | CIRAD | Landrace |
| *Musa ornata* | AA | USDA-ARS | Wild |
| Pahang *M. acuminata* CMR | AA | CIRAD | Wild |
| Pisang liling | AA | CIRAD | Landrace |
| Cavendish Grand Naine | AAA | Rahan Meristem | Cultivar |
| Cavendish- GCTCV-247 | AAA | Queensland | Cultivar (somaclone) |
| Cavendish-Formosana | AAA | Queensland | Cultivar |
| Cavendish-Williams | AAA | Queensland | Cultivar |
| FHIA-25 | AAA | Queensland | Hybrid |
| Lacatan | AAA | USDA-ARS | Cultivar |
| CRBP 39 | AAAB | USDA-ARS | Hybrid |
| FHIA-18 | AAAB | Queensland | Hybrid |
| FLF | AAAB | Queensland | Hybrid |
| PV-42-320 | AAAB | USDA-ARS | Hybrid |
| Cachaco | AAB | Bioversity | Cultivar - Bluggoe |
| Pisang Lawadin | AAB | Bioversity | Landrace |
| X-17 | ABA | CIRAD | Hybrid |

**Supplemental Table 2.** Characteristics of the TR4 (*Fusarium odoratissimum)* – banana interaction experiments for each investigated inoculation method.

| ***Method^1^*** | ***Inoculum dose*** | ***LP^2^*** | ***FST^3^*** | ***Chlorosis*** | | ***Corm***  ***Disc%*** | ***DI^4^*** | | ***Corm dry***  ***Weight^5^*** | ***Ct values*** |
| --- | --- | --- | --- | --- | --- | --- | --- | --- | --- | --- |
|  |  |  |  | ***%*** | ***Class*** |  | ***%*** | ***Group*** |  |  |
| DM + Soil | 1 x 10^3^ spore ml^-1^ | 3 | 10 | 54.1^B^ | III | 17.3 ^A^ | 36 ^BC^ | I | 8.3^G6^ | 21.19 ^DEF^ |
|  | 1 x 10^4^ spore ml^-1^ | 2 | 7 | 91.7 ^CD^ | IV | 76.7 ^CD^ | 96 ^E^ | III | 2.1^ABCD^ | 19.38 ^CDE^ |
|  | 1 x 10^5^ spore ml^-1^ | 2 | 5 | 100 ^D^ | IV | 91.7 ^D^ | 100^E^ | III | 1.0^AB^ | 18.77 ^BCD^ |
|  | 1 x 10^6^ spore ml^-1^ | 2 | 5 | 100^D^ | IV | 88.3^D^ | 100 ^E^ | III | 0.7^A^ | 18.61 ^ABC^ |
| DM + Sand | 1 x 10^3^ spore ml^-1^ | 3 | 10 | 40.7 ^AB^ | II | 1.5 ^A^ | 8 ^A^ | I | 6. 4^EFG^ | 24.71 ^GH^ |
|  | 1 x 10^4^ spore ml^-1^ | 3 | 10 | 41.8 ^AB^ | II | 6.1 ^A^ | 24 ^AB^ | I | 6.6^EFG^ | 24.73 ^GH^ |
|  | 1 x 10^5^ spore ml^-1^ | 3 | 6 | 91.7 ^CD^ | IV | 57 ^BC^ | 92 ^E^ | III | 2.6^D^ | 15.81 ^AB^ |
|  | 1 x 10^6^ spore ml^-1^ | 2 | 7 | 100 ^D^ | IV | 89.1 ^D^ | 100 ^E^ | III | 1.4^ABCD^ | 16.98 ^ABC^ |
| PM + Soil | 1 x 10^3^ spore ml^-1^ | 3 | 10 | 31.0 ^A^ | II | 3.5 ^A^ | 20 ^AB^ | I | 7.3^FG^ | 24.20 ^FGH^ |
|  | 1 x 10^4^ spore ml^-1^ | 3 | 10 | 41.6 ^AB^ | II | 7.4 ^A^ | 34 ^AB^ | I | 6.6^EFG^ | 25.05 ^H^ |
|  | 1 x 10^5^ spore ml^-1^ | 3 | 10 | 57.1 ^AB^ | III | 20.5^A^ | 62^CD^ | I | 6.1^EFG^ | 22.43 ^EFG^ |
|  | 1 x 10^6^ spore ml^-1^ | 3 | 6-10 | 77.3 ^B^ | IV | 45.99^B^ | 88 ^D^ | III | 4.6^EFG^ | 19.74 ^DE^ |
| CM + Soil | 2.5 g / L^-1^ soil | 2 | 6 | 76.7 ^C^ | IV | 46.0^B^ | 80 ^D^ | II | 5.6^EF^ | 18.29 ^ABC^ |
|  | 5 g / L^-1^ soil | 2 | 7 | 95.0 ^D^ | IV | 82.1 ^CD^ | 96 ^E^ | III | 2.2^BCD^ | 16.44^ABC^ |
|  | 10 g / L^-1^ soil | 2 | 8 | 95.0 ^D^ | IV | 60.5^BC^ | 96 ^E^ | III | 2.5^CD^ | 15.06 ^BCD^ |
|  | 20 g / L^-1^ soil | 2 | 5 | 100^D^ | IV | 89.5 ^D^ | 100^E^ | III | 1.0^ABC^ | 15.32 ^BCD^ |
| KM + Soil | 3 kernel/ L^-1^ soil | 4 | 10 | 48.8 | II | 20.6 | - | I | - | NI |
|  | 6 kernel/ L^-1^ soil | 4 | 10 | 39.8 | II | 4.6 | - | I | - | NI |
|  | 10 kernel/ L^-1^ soil | 3 | 10 | 43.5 | II | 12.2 | - | I | - | NI |
|  | 20 kernel/ L^-1^ soil | 3 | 10 | 42.6 | II | 11.8 | - | I | - | NI |
| Controls | DM/Soil/water | 1 | 10 | 16.2 | I | NA | - | - | 8.3 | - |
|  | DM/Sand/water | 1 | 10 | 15.7 | I | NA | - | - | 8.2 | - |
|  | PM/Soil/water | - | 10 | 19.0 | I | NA | - | - | 7.7 | - |
|  | Mock^7^ | - | 10 | 13.7 | I | NA | - | - | 5.2 | - |

1. DM: dipping method soil, DMS: dipping method sand, PM: pouring method, CM: chlamydospore method, KM: kernel method. 2. Latency period (LP): week in which at least one plant started to show external symptoms. 3. Final sampling time (FST), weeks after inoculation in which half of the plants were harvested. 4. Disease Index (DI). 5. Corm dry weight of the entire corm in grams. 6. Significance, values within a column followed by the same letter were not significantly different according to LSD test at P=000.1. 7. Untreated plant. NA= not applicable; NI = not included; - not measured.
